# A New Web-Based Tool for RTO-Focused Animal Shelter Data Analysis

**DOI:** 10.3389/fvets.2021.669428

**Published:** 2021-05-25

**Authors:** Tom Kremer

**Affiliations:** Minerva Schools at KGI, San Francisco, CA, United States

**Keywords:** RTO, return to owner, data visualization, animal shelter, stray dogs, geographical/spatial analysis, web application, dog

## Abstract

Animal shelters are increasingly interested in reducing their intake and helping their communities keep and care for animals. Improving Return-to-Owner (RTO) rates of stray dogs is one path to save significant shelter space, time, and costs and keep animals with their caregivers and communities. Aggregating and visualizing RTO data spatially are useful for identifying trends and highlighting areas for potential interventions. Since shelters collect similar data, an interactive web application was developed to make such an analysis easily reproducible. This paper presents the tool's capabilities via a case study of 2019 data from the Dallas Animal Services shelter, covering the relationship between stray intake and RTO rate, the distances traveled from home by RTOed strays, microchip use across the city and its relationship with RTO rate, and the length of stay of RTOs and other outcome groups. Findings include showing that 70% of RTOed strays traveled at most 1 mile away from home and 42% up to block away, and that at-large, adult strays that had a microchip had a 71% RTO rate compared with 39% without one. The results affected the shelter's hold time for strays, highlighted target areas for microchip programs, and motivated neighborhood-based methods to locate found dogs' owners. Shelters are welcome to use the tool and participate in the development of new analytical lenses and visualizations that would best suit their needs.

## Introduction

Animal shelters take two approaches in measuring and evaluating their services. The first is looking at their outcomes, usually in terms of live release rate, and improving it through various programs (1–4). Many shelter-level studies conducted with academia and animal welfare organizations examine trends or interventions targeted at improving outcomes (2, 5). This should not come as a surprise, because a high live release rate is a helpful performance indicator for any shelter. The second path is to examine and reduce intakes rather than improve outcomes (6). This has been the focus, for example, of spay–neuter programs (7, 8).

This perspective can be framed within a broader re-evaluation of the shelter's role. Human Animal Support Services (HASS), a coalition of shelters and animal welfare organizations across the US, tries to rethink the role and structure of shelters by building programs that help keep animals within their community, with the shelter primarily functioning as an emergency medical care and short-term housing center for pets in urgent need (9). An emphasis on understanding and reducing intakes is essential within this framework. However, even without such repurposing of the shelter, focusing on intake prevention supports shelter's interests [for an example with cat populations, see (10)].

One key activity that can promote this goal is to improve Return-to-Owner (RTO) rates. The rate refers to the fraction of stray intakes that are returned to their owners by field officers or after a short stay at the shelter (11). RTO has significant benefits: taking in a stray, desexing it (as mandated in most states), caring for it, and rehoming it is more costly and time-consuming than returning it to its owner, while in the meantime, it also takes shelter space, which means that the shelter can help fewer animals over time (12). Reuniting pets with their owners also relieves the distress caused by a lost pet and contributes to the trust in the shelter within its community (11).

Improving RTO rates has been recognized in the past as an underutilized means to decrease euthanasia rates, and a look at nation-wide shelter statistics suggests that there is a large room for improvement (6, 13). As could be expected, cats have much lower RTO rates than dogs, partly because many cats taken in have no homes to return to. While there is certainly room for improving cat RTOs, this study looked only at dogs, and thus so do all data, tables, and figures hereafter. Naturally, focusing on RTOs is relevant for shelters in which strays make up a significant portion of their intakes. For a rough estimate of the number of stray dogs and RTO rates across US shelters, Table 1 summarizes this information based on 3,226 organizations that reported their 2019 calendar year data to Shelter Animal Counts (SAC) (13).

**Table 1:** Intakes, strays, RTOs (in thousands), and RTO rates as reported to SAC, 2019.

**Organization type**	**Intakes**	**Strays**	**RTOs**	**RTO rate (RTOs/strays)**
Government animal services (*n* = 460)	968.3	636.6	247.2	39%
Shelter/rescue, govt. contract (*n* = 425)	598.3	295.7	129.8	44%
Shelter/rescue, private (*n* = 2,341)	725.4	119.4	36.3	30%
Total (*n* = 3,226)	2,292	1,051.7	413.3	39%

Overall, 46% of all reported dog intakes were strays (1.081 million out of a total intake of 2.292 million dogs), which was the leading intake type, followed by about 500,000 owner surrenders. Of these strays, the RTO rate across all reporting organizations was 39%. Looking at the subcategories of organizations as listed on SAC, RTO rates were 39% for governmental animal services, 44% for shelters or rescue with government contracts, and 30% for private shelters and rescues. These rates may be higher than the actual RTO rates, since they capture all RTOs and not only out of stray intakes, which includes confiscated dogs and owner surrenders. These data suggest that while the additional RTO potential might vary between organizations, there were at least 600,000 strays that were not returned to their owners.

Of course, some of these dogs could not be returned to their owners, because they were abandoned by them or did not have any. Another insight into the RTO gap can be drawn from a study conducted by Weiss et al. that surveyed owners on their lost pets (14). They estimated that 15% of dogs run away or get lost at least once, and that about 766,000 dogs are never reunited with their owners each year. Presumably, many of them end up in local shelters. Even if only half of the extra 600,000 intakes from 2019 are lost pets, when considering the cost, time, and shelter space taken for the care, desex, and rehoming of each animal, as well as the psychological and community-building benefits, the above estimates suggest that improving RTOs is a desirable goal for many shelters.

With this general motivation to study RTOs in mind, this research effort began by analyzing stray intake and RTO data from the Dallas Animal Services (DAS) shelter, aiming to illuminate questions that would support the shelter's effort to improve its RTO rates. In the fiscal year of 2019, 30,659 dogs were admitted into the shelter of which 20,738 (68%) were strays, and of these, 10,015 (48%) were RTOs. As suggested above, data about abandoned and free-roaming dogs would be relevant to assess the additional RTO potential in Dallas, i.e., how many of the 52% of strays not RTOed could be had the owner was found. Unfortunately, no such data were available. Physical condition could be one proxy for identifying whether an intake with no indication of an owner has RTO potential, but 95% of these intakes were similarly labeled as healthy. The DAS staff, through their communications with community members and local partners, assumed that they would have known of a large free-roaming dog population or recurring abandonments of pets; they believed that a meaningful part of the non-RTOed strays had owners to get back to who for a variety of reasons did not contact the shelter or provide their dogs with a form of identification. Thus, despite this imperfect knowledge, a dive into their data could help clarify how their intake and RTO patterns improve their RTO-related policies and programs.

The following questions were chosen with the shelter staff to guide this study:

1. What is the relationship between the number of strays and RTO rate per ZIP code?
2. How far do RTO strays travel away from home? Does that vary based on the stray's found location?
3. How long do strays stay before they are RTOed? Does length of stay (LOS) vary based on the owner's address?
4. What is the difference in RTO rate between strays found with or without a microchip? Were microchips uniformly present across the city?

As evident from these questions, a spatial analysis was appropriate to examine stray and RTO data. Mapping the data would allow the shelter staff to examine the summary statistics and trends in relation to different parts of the shelter's jurisdiction. While there are no previous spatial studies of stray dog intakes and RTOs specifically, several studies used Geographic Information Systems (GIS) to target interventions aimed at stray dog and cat population. Miller et al. used GIS to select areas for intervention targeted at preventing euthanasia by reducing cat and pit bull intakes (15). They built maps on both ZIP level and Census tract levels that facilitated the selection of intervention areas for intake reduction and applied an intervention program made up of free spay/neuter surgeries, behavior trainings, vaccines, and retail gift cards, among others. They found that the spatial approach was valuable in selecting a target for intake reduction intervention as well as tracking its success. Spencer et al. used GIS to map the intake patterns of a shelter in Florida, identify areas with high stray dog intake, and investigate the reasons for the high intake through interviews with residents (16). Spindel et al. used intake locations of dogs identified with two types of canine viruses and their antibodies to target vaccination programs, and Sokolow et al. used GIS to track the spatial distributions of diarrheal disease among dogs in a northern California animal shelter (17, 18).

Other studies used GIS to characterize stray dog and cat population and study links between their pattern and sociodemographic indicators. One such study used geolocations of adoption outcomes from an animal shelter in Massachusetts on a Census-block level to investigate the link between adoption and both distance from the shelter and demographic indicators, such as median household income (19). In another study, cat intakes and deaths were geocoded and examined across Census tracts in Boston for their correlation with human premature death and socioeconomic indicators reflecting depravation (20). Outside the United States, one study mapped stray dog population in São Paulo, Brazil to evaluate the association of local sociodemographic and environmental factors with the population perception of the presence of free-roaming stray dogs (21). The researchers used districts as the geographic unit studied, spanning between 63 and 151 km^2^, larger than Census tracts but smaller than ZIP codes. Similarly, Reading et al. identified clusters of owner addresses from which cats were surrendered to shelters (22). They were interested in specific addresses or blocks and, thus, used addresses to construct a density map and a clustering analysis. Aguilar and Farnworth studied stray cats in Auckland, New Zealand (23). They processed exact intake locations and identified high density areas with stray cats and reported their results in the neighborhood level around the Auckland region.

This study used ZIP codes as the main geographical hierarchy to characterize stray intake, as motivated in the Methods section, while also focusing on the spatial dimensions of RTO rates and microchip prevalence to explore a potential for program improvements.

The examination of RTO rates among microchipped and non-microchipped dogs builds upon several past studies. Lord et al. studied 3,425 stray dogs from 53 shelters, excluding Field RTOs for which no microchip data were available, and found that the median RTO rate across studied shelters for microchipped dogs was 52%, compared with an overall RTO rate of 22% (11). A similarly large study in Queensland, Australia examined microchip registration and RTO rates among 7,258 adult stray dogs and found an 80% RTO rate for microchipped dogs, including those with missing or faulty data, compared with 37% RTO rate for dogs without a microchip (24). A study in Czech Republic examined 10 years of shelter data, 5 years before and after a mandatory microchip decree was put in place in 2009 (25). In addition to finding that more dogs had microchips in the period after the decree, and that RTO among those with a microchip has slightly increased, their reported data showed that over the entire study period, microchipped dogs had a 77% RTO rate (1,056/1,379) compared with 42% (1,295/3,076) for non-microchipped dogs. Studies with smaller sample sizes (in the hundreds) in Spain and Serbia have also found similar differences in RTO rates (26, 27). This study builds upon these previous results by examining microchip presence across different areas of the shelter's jurisdiction. Furthermore, since microchip practices may differ between countries, this study provides an additional replication for the results of Lord et al. for a US-based shelter.

To enable more shelters to analyze their data based on the guiding questions identified above, a web-based interactive dashboard, temporarily named “Shelter Databoard,” was built to visualize the results of the analysis. Information systems used by shelter do not natively offer this type of analysis, and since the data collected for this analysis are collected by many other shelters, the tool was built to take in a CSV file that any shelter could export from its information system.

In this paper, I will dive into the analysis of the DAS data as a case study to highlight the tool's capabilities and the insights that can arise from looking at shelter-level data this way. The Methods section provides additional context on DAS and goes through the data fed into the tool and the methods used to derive the different visualizations, which are then presented in the following Results section by the four research questions. I then discuss some of the tool's implications on DAS's practices and potential insights that may arise for different shelters and end with a brief overview of future directions, including the incorporation of Census tract data and an invitation for shelters to use the tool. A link to a live version of the tool with DAS data, courtesy of the shelter to share it, appears in the Future Research section.

## Methods

### Dallas Animal Services

DAS is the government-operated municipal animal shelter for the city of Dallas, Texas and provides public safety and animal care services to residents of Dallas. It takes in any pet in need, regardless of space, and is located at ZIP code 75212. According to the US Census, the human population in Dallas in 2019, the year covered by the data, was 1,343,573 (28). Stray dogs are defined by DAS as any dog found in the field or brought in by a person who is not the dog's owner, as opposed, for example, to owner surrenders, incoming transfers, and dogs taken in for custody, quarantine, and following an eviction or cruelty. RTOs are accomplished in two ways: Field RTOs occur when the owner is found by a field officer, and Shelter RTOs occur when a dog was RTOed after it was brought into the shelter. References to RTO across the paper include both categories, unless explicitly mentioned; for example, the distance traveled by RTOs includes both categories, whereas LOS does not apply to Field RTOs.

A few additional details on RTO procedures in DAS can provide further helpful context. First, DAS has a stray hold period policy that defines different hold times across age and available identification. Puppies under 4 months have no hold period; those 4–6 months have a 1-day hold; older dogs without any form of identification (such as a microchip or a collar) are held for 3 days, and adult dogs for whom identification is present have a 5-day hold period. Dogs taken in for custody or quarantine face longer hold periods but were not included under the scope of this study. Second, adult dogs (over 6 months) must go through desexing and microchipping under the shelter's ordinance, unless deemed unfit for surgery. Field RTOs are exempt from both requirements. Finally, DAS's policy requires owners to pay reclaim fees for Shelter RTOs, associated with the stay, microchipping, and desexing procedures. In practice, however, the fees are commonly waived, based on the owner's needs and at the shelter's discretion.

### Data Preparation

All cleaning and analysis were performed in R (29). DAS provided the dataset for this case study, which consisted of all dogs that have gone through the shelter in the 2019 Dallas fiscal year, October 1st, 2018 to September 30th, 2019, as pulled out of its information system in the beginning of this research. For each dog, the following features were used: intake and outcome dates, LOS (the number of days between these dates plus one), intake type (e.g., stray, owner surrenders), intake subtype (whether there was any indication of an owner, e.g., a collar), intake condition (e.g., healthy, injured, sick), breed, age, microchip scan result (yes, no, or unknown, regardless of registration or correctness of details), intake address and ZIP code, outcome type (e.g., adoption, RTO), and outcome address and ZIP code. The intake address and ZIP code for stray dogs were their found location (also known as Crossing), whether they have been brought in over the counter or RTOed by a field officer. Outcome address for RTOs was the owner's address, existing (RTO) or new (adoption). Breed was only used when examining microchip prevalence, as discussed later; since it is a notorious field due to people's inability to accurately identify dog breeds, only pure vs. mixed breed status was considered (30). The final data file contained a single record for each instance of a dog entering the shelter, which means that some dogs appeared multiple times if they re-entered the shelter.

This analysis used ZIP codes as the main geographical hierarchy that organizes results for several reasons. First, it was available for all data points. Second, the high-level overview of the geographical patterns around strays and RTOs that is obtainable via ZIP codes was sufficiently relevant for the shelter. Finally, the shelter staff are used to working with ZIP codes in their daily work and when compiling different metrics. Nevertheless, ZIP codes have clear limitations—as suggested by Reading et al., they are too wide to support targeted interventions and cannot be correlated with demographic data. Future research to meet these limitations is discussed later.

Due to the geographical focus, 50 dogs that were missing an intake ZIP code were removed, leaving a total of 30,609 dogs in the final dataset. Stray dogs whose intake address was listed as the shelter's address (*n* = 205) were excluded from the stray count to avoid skewing the results, as they are essentially missing their true found location. After this filtering, it was still clear that most of the shelter's intake comes from strays (*n* = 20,763), which motivated the shelter's interest in its RTO patterns. RTO rate was defined as the number of RTOs out of the number of strays, which for the shelter stands at 48% (10,035/20,763). Scarlett (6) suggested that this is a conservative definition because stray puppies are less likely to have owners and could be excluded from the calculation, as RTO should only be about strays that have owners. Yet in DAS's case, the RTO rate for puppies was 18% (381/2,091), which was found high enough to include.

The second research question, investigating the distance RTOs travel away from home, required manual inspection to ensure data integrity. The distances were derived as follows: first, data were filtered to remove dogs (*n* = 4,778) that had identical intake and outcome addresses. According to the shelter staff, this happened often when field officers used the shelter's or the owner's address instead of the location in which the dog was found. Then, the distance traveled by each dog was calculated in two ways: first, using the intake and outcome addresses as is to calculate a walking distance *via* Google Maps API and second, by geocoding the address and then calculating the distance between them *via* the *Imap* package, which finds the geodesic distance between two points specified by latitude–longitude pairs. A manual examination of the two types of distance searches by the author found that the first method, using the explicit addresses, was more error prone, including erroneous distances and NA responses, so the *Imap* approach was chosen. Since the *Imap* address also indirectly used the addresses for geocoding, the results were further examined to identify wrong identifications resulting from faulty data. This way, for example, data with missing letters were corrected, and addresses that exist in multiple states were modified to similar ones in Dallas. In the cleaning process, distances for 8 dogs were tuned, and 80 were removed, 2 of which due to unclear addresses and 78 due to owner addresses outside Texas that resulted in over 25 miles traveled (cut-off chosen arbitrarily). The shelter's geolocation was also found to center the maps, and a spatial file containing the boundaries of all ZIP codes was prepared for the spatial visualizations.

### Data Analysis

Starting with question #1, examining the relationship between the number of strays and RTO rate, these two quantities were calculated by aggregating intake and outcome data for each intake ZIP code. To visualize the results as a choropleth, in which each ZIP code is colored by the quantity of interest, a spatial file containing the ZIP code boundaries for Dallas was obtained. This spatial file was presented on top of a base map centered around the Dallas City Hall obtained *via* the Google Maps API. ZIP codes with <10 strays were excluded.

For question #2, looking at the distances traveled, after the data were prepared as described above, the distribution of distances traveled by the dogs with different intake and outcome addresses (*n* = 5,228) was plotted on a histogram, and summary statistics were obtained. The distances were also aggregated by the found ZIP codes and plotted as before, to identify the trends in different parts of the city.

Question #3 regarding LOS relied on the LOS feature available in the data, but 5 days was deducted from it to account for the stray hold period for adult dogs at DAS. To create a less noisy comparison with other outcome groups, only stray adults that were found “at-large,” i.e., without any indication of an owner (as opposed to others labeled as “possibly owned” or “confined” under the intake subtype field) and had no health condition, were examined. For RTOs, only Shelter RTOs were counted. It is possible to create a more sophisticated comparison between dogs that are similar on more characteristics (i.e., not only age, subtype, and condition) or more closely similar (e.g., account for exact age). However, since this comparison is not meant to provide a comprehensive model for LOS but a rough estimate of its difference across outcome groups, this one suffices. The distributions, median, and 90th percentile of post-hold LOS were thus compared between Shelter RTO (*n* = 2,400), adoption (*n* = 3,916), and transfer (*n* = 1,210). Spatial plotting was done similarly to previous sections, but this time aggregated by outcome rather than intake ZIP code to examine the LOS for RTOs across the city.

Finally, to investigate the microchip layer of the data for question #4, stray and RTO counts were found for dogs with and without microchips, excluding those with unknown status. Similar to LOS, a simple comparison between “microchip” and “no microchip” could be misleading, because there could be other differences between the groups that might affect the different RTO rates. The “no microchip” group was identified to have more puppies than the microchip group, and they are much harder to RTO. The microchip group also had more strays that were marked with an intake subtype of “possibly owned,” meaning that there was a potential indication of an owner, e.g., a tag or word of mouth. They are easier to RTO, regardless of a microchip. Thus, only healthy adults that were found “at-large” (*n* = 13,794) were divided by microchip status, and RTO rates were compared through a chi-square analysis of a 2 × 2 contingency table.

As a final consideration, it could be that the non-chipped dogs lived in ZIP codes that had lower RTO rates for other reasons, such as shelter accessibility. To account for intake location, RTO rates between “microchip” and “no microchip” groups were compared seven times using a chi-square analysis when only selecting the healthy at-large adults from each of the highest-intake strays identified under question #1, the smallest of which recorded 1,015 strays.

Other available variables, such as color, breed, and date of intake, were similarly distributed among dogs with and without microchips, suggesting that they do not account for the difference. Again, it is possible to create a more sophisticated comparison between dogs of all subtypes that are similar on more characteristics than those used above, but since achieving an RTO is most relevant for dogs with this profile (i.e., adults with no owner indications) and most strays in the data fell under the “healthy, at-large, adult” description, a direct comparison was performed between these groups.

Then, for each ZIP code, a “microchip rate” was defined as the number of microchipped strays found in that ZIP code out of all strays found in it. This rate was plotted against the ZIP code's size, to examine whether larger-intake areas also had more microchip awareness. Finally, the microchip rate was plotted on a map as in previous sections.

The web-based Shelter Databoard visualizes the result of the analysis, built using the *Shiny* R package. The tool takes in the preprocessed CSV file with shelter data as described above. Some additional settings are manually tuned to enhance readability (for example, legend values). The user can control the date range of data fed into the figures to compare different periods, switch between types of data on an interactive map, break down the data by different dimensions (for example, examine only over the Shelter or Field RTOs), and find key summary statistics of their data. As of writing this paper, new sections were added to the tool, including demographic data and a visualization of euthanasia requests, as discussed in the Future Research section.

## Results

### The Relationship Between the Number of Strays and RTO Rate per ZIP Code

**Figure 1:**
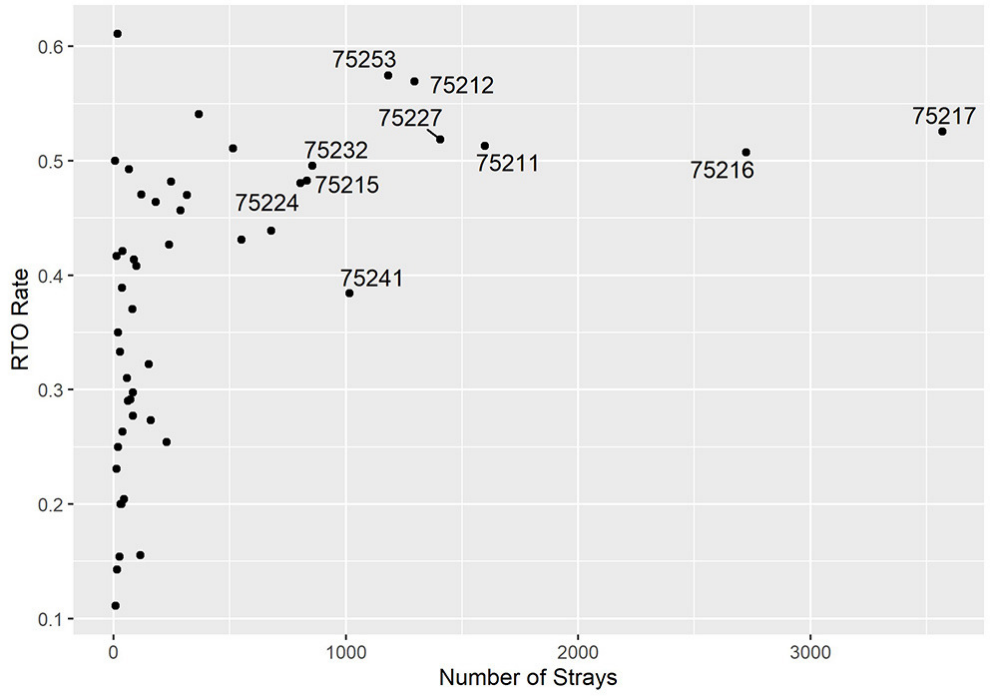
RTO rate and the number of strays per ZIP code. Labels indicate high-intake areas.

Most ZIP codes contribute a small share of the shelter's stray intakes, whereas a few ZIP codes have high intakes, as shown in the horizontal axis of Figure 1. While the ZIP codes with smaller intakes display high variability of RTO rate (vertical axis), the few large areas have roughly similar ones around the 50% mark. ZIP code 75241 stood out with a high-intake count but lower than usual RTO rate at 38%. When looking at Field RTOs only (who never arrived at the shelter; not plotted), the trend looked similar.

**Figure 2:**
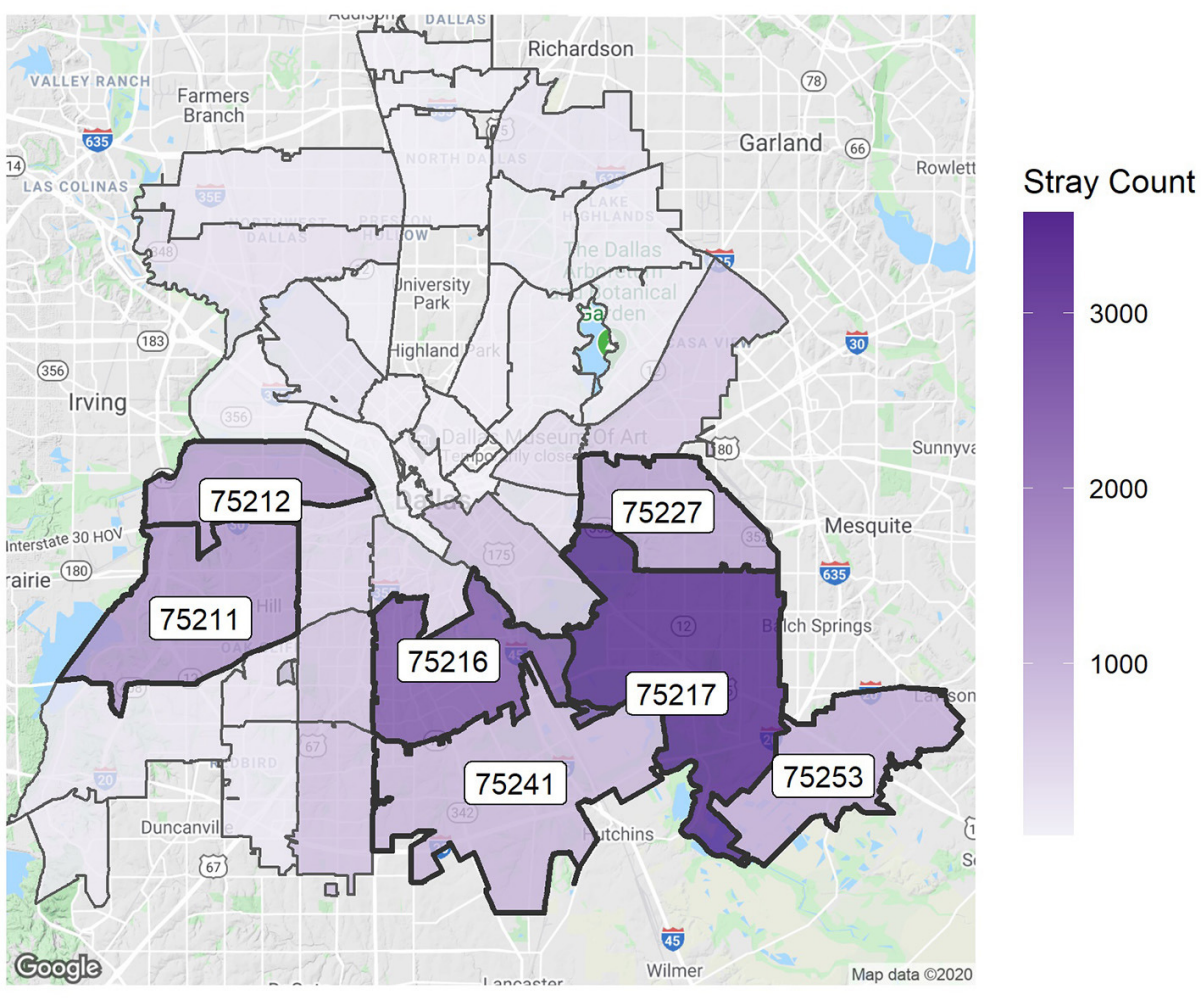
Strays per ZIP code. The map is centered on Dallas City Hall. The seven largest areas are labeled.

**Figure 3:**
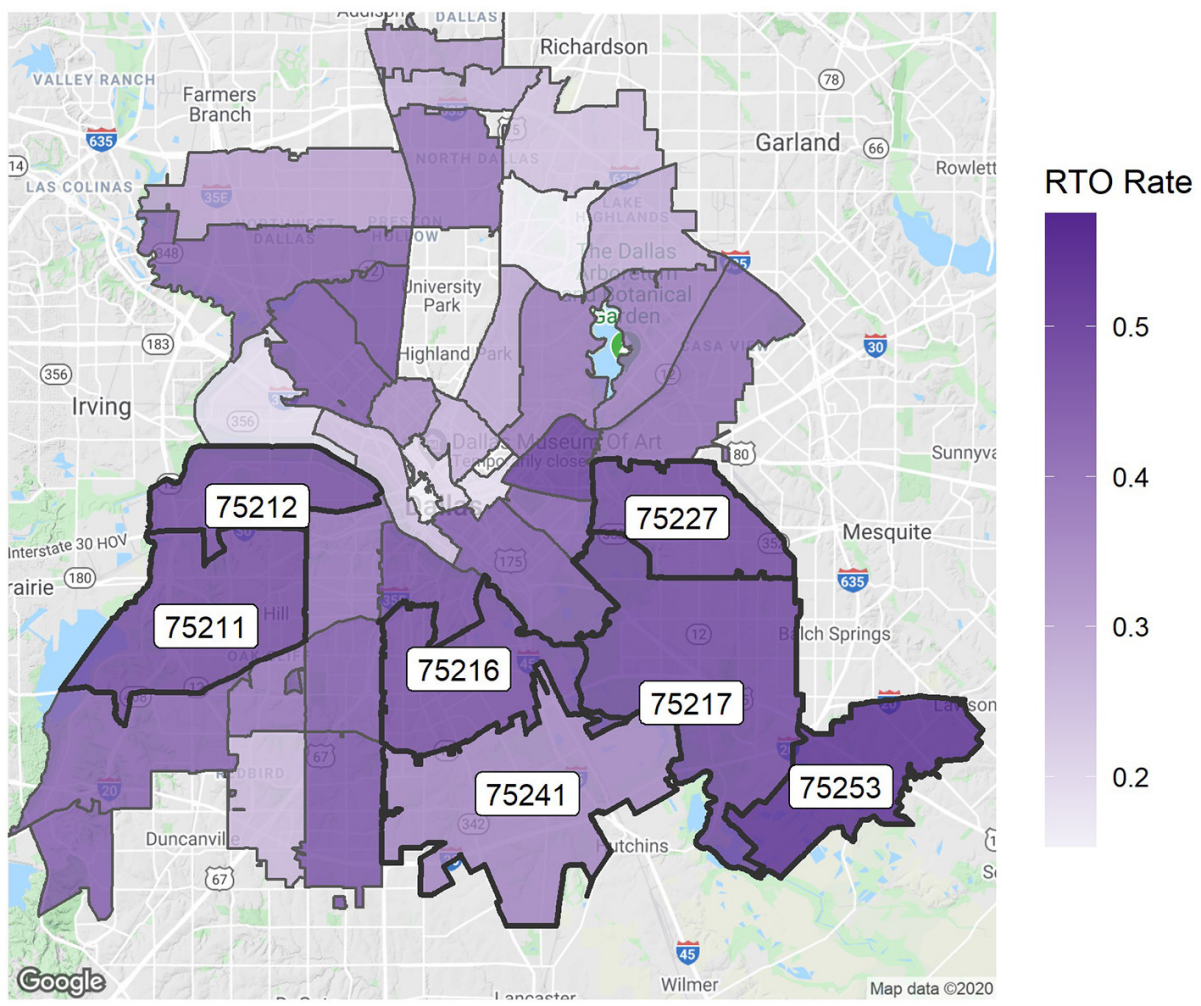
RTO rate per ZIP code. The seven highest-intake ZIP codes are labeled.

It is also helpful to see the dimensions of this figure on a map of Dallas. Figure 2 visualizes the horizontal axis of the previous figure—the number of strays across different ZIP codes. Moreover, 63% of all strays come from the seven labeled ZIP codes. The map clearly shows that most strays are found in the southern area of the city. Similarly, Figure 3 shows the vertical axis of Figure 1—the RTO rate for each ZIP code. Generally, the southern areas with higher stray numbers also have high RTO rates compared with the northern regions, but the variability is not as strong as in the number of strays.

### How Far Do RTO Strays Travel Away From Home?

**Figure 4:**
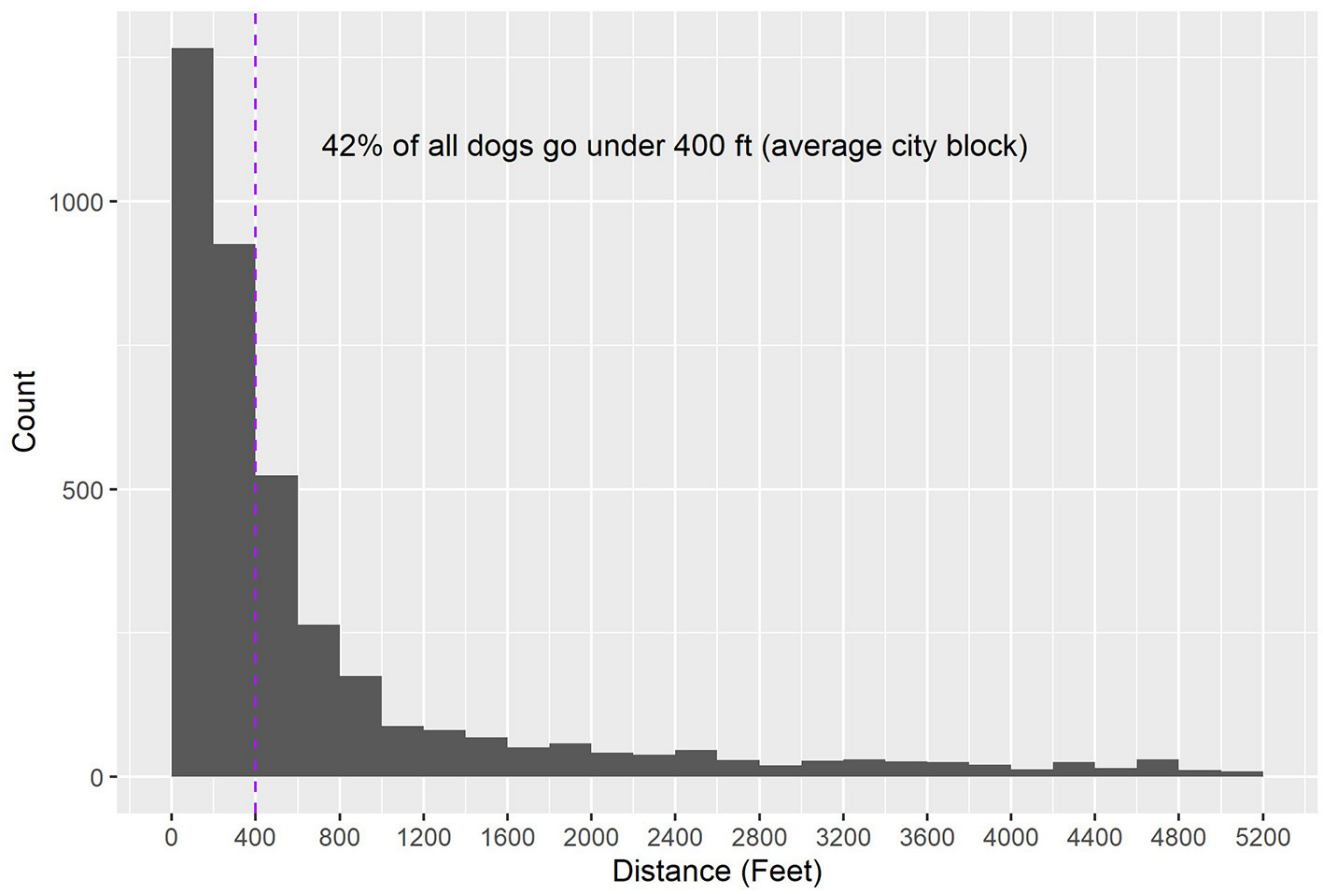
Distance traveled by RTO strays, excluding those with identical intake and outcome addresses, zoomed in on dogs who traveled up to 1 mile. The 400 ft mark is indicated with a purple line.

As mentioned before, out of 10,000 RTO strays with known owner addresses, 4,775 had the exact same owner address and found location. Out of the other 5,228, 70% of dogs are not found beyond 1 mile away from their owner address. Figure 4 zooms into the 70% of dogs that walk under 1 mile. Of these 70, 60, or 42% of all dogs, go <400 ft away from their owner address (an estimate of an average city block).

**Figure 5:**
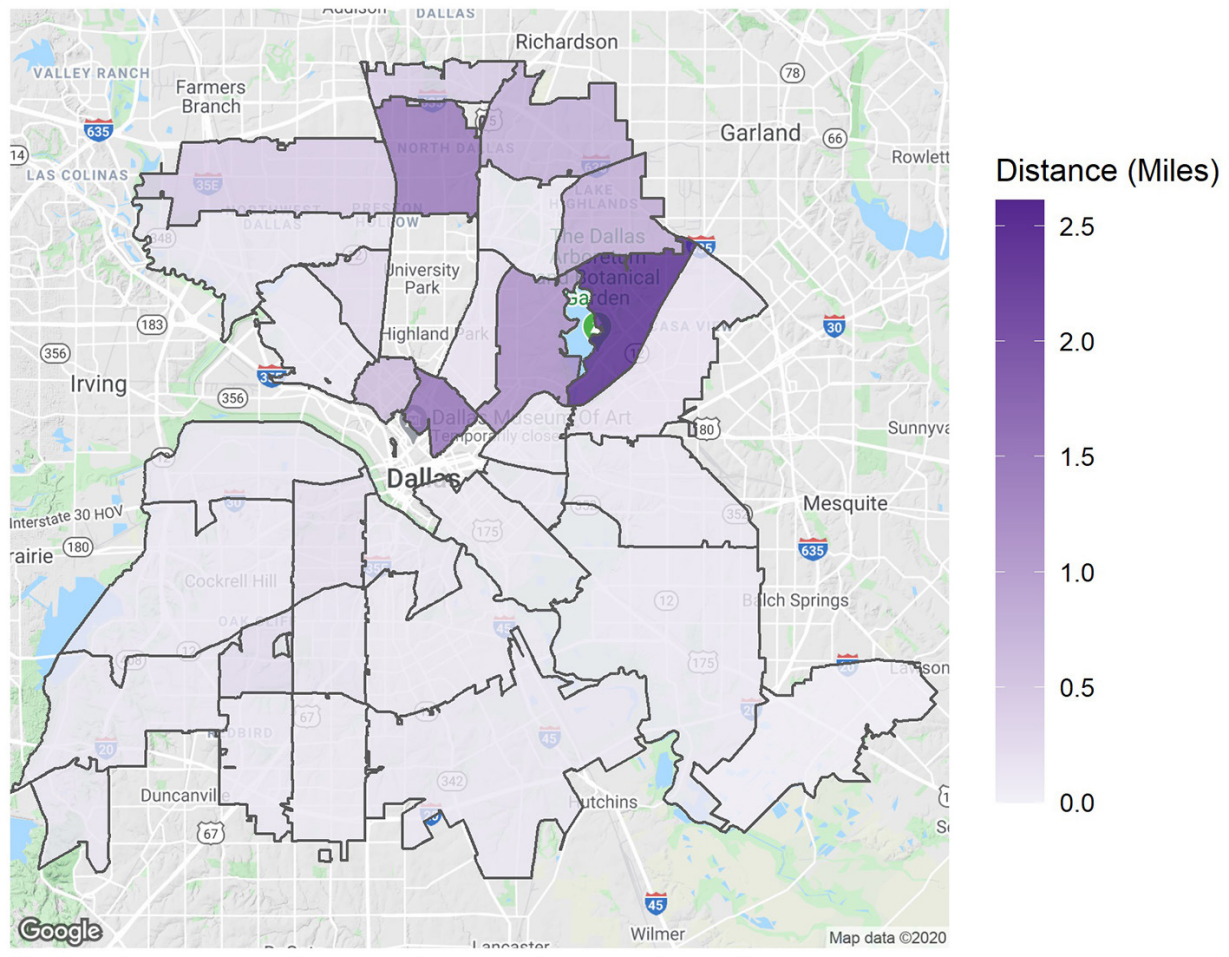
Median distance traveled by RTO strays per intake ZIP code.

The results so far were aggregated for the whole shelter, but a further question was whether there was some variation in these distances for dogs found in different locations. In other words, are dogs found in some parts of the city likely to have gone farther from home than others? Figure 5 tries to answer this question by showing the median distance traveled (in miles) by all dogs found in a certain ZIP code. Dogs found in the northern part of the city tend to travel farther away from home (1.5–2.5 miles) than those in the southern ZIP codes (around 0–0.5).

### How Long Do Strays Stay Before They Are RTOed? Does That Vary Based on the Owner's Location?

**Figure 6:**
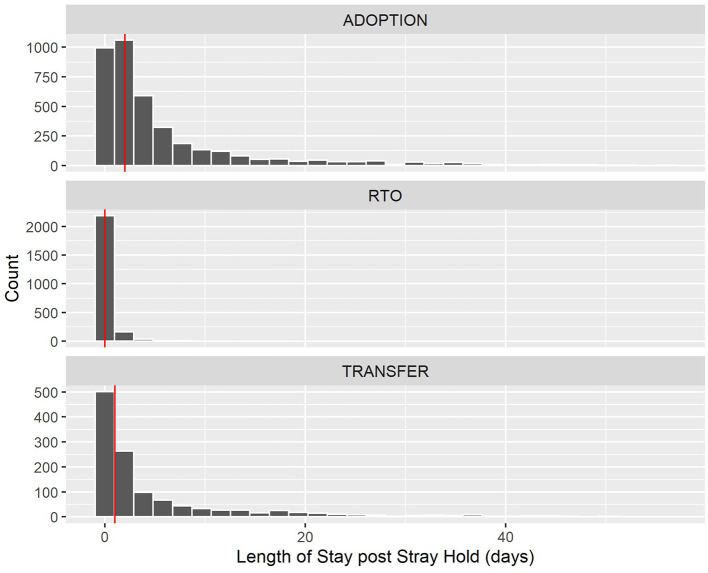
Length of stay after hold period for healthy, adult stray dogs who were adopted, RTOed, or transferred. The red vertical line indicates the median for that outcome type, also summarized in a table below.

Figure 6 shows that post-hold period LOS for RTOs is much lower than adoptions and transfers. All outcome categories exclude the upper 4–5% outliers of their outcome types with a cut-off of 60 days to allow an easier view. Moreover, 91% of dogs were reclaimed during the 5-day hold stray period; hence, the median and 90th percentile values of 0 were post-hold LOS. For adoptions, while the median post-hold LOS was 2 days, there was a longer “tail” into the longer stays area, with 24% of dogs staying at least 7 days, after which the number of days decays until hitting the 90% mark at 16 days. Transfers were similar to adoptions, with a lower median of 1 day, a similar 23% of dogs that stayed a week or more, and a slightly higher 90th percentile at 17 days. Table 2 summarizes these summary statistics for each outcome category for comparison. The low RTO statistics compared with other live release outcome types help to demonstrate the additional days a stray dog is expected to spend in the shelter if not RTOed.

**Table 2:** Summary statistics of LOS post a 5-day hold period (in days) for different outcome types and the percentage of dogs who stayed at least a week per group.

**Outcome type**	**Count**	**Median LOS**	**90th percentile LOS**	**LOS ≥7 (%)**
RTO	2,400	0	0	0
Adoption	3,916	2	16	24%
Transfer	1,210	1	17	23%

Focusing back on RTOs across Dallas, few differences (of <1 day) were observed in the mean, median, and 90th percentile of LOS when grouped by Owner ZIP codes. In other words, LOS for Shelter RTOs was relatively unaffected by the area in Dallas in which the owner lives.

### What Is the Difference in RTO Rate Between Strays Found With or Without a Microchip? Were Microchips Uniformly Present Across the City?

In a naïve comparison, excluding 2,013 strays whose scan status was unavailable, those found with a microchip were RTOed 70% of the time, compared with 33% when no microchip was present. When comparing only healthy, at-large, adult strays with and without a microchip, the gap has narrowed slightly but was still meaningful and statistically significant: 71% RTO rate for strays with a microchip compared with 39% for non-microchip (Table 3; χ^2^ = 1, 101, *df* = 1, *p* < 0.001). The increase in the non-microchip rate was likely due to the exclusion of puppies and “possibly owned” strays.

**Table 3:** RTO rates for all strays and healthy, at-large, adult strays with and without microchips.

**Which strays**	**Microchip**	**Strays**	**RTOs**	**RTO rate**
All	No	13,032	4,265	33%
All	Yes	5,691	3,971	70%
Healthy at-large adults	No	8,311	3,213	39%
Healthy at-large adults	Yes	3,867	2,744	71%

When performing the test again but only selecting the healthy at-large adults from each of the highest-intake ZIP codes, the RTO rates remained almost the same, varying between 39 and 45% for “no microchip” and 71 and 75% for “microchip,” and the difference was similarly statistically significant (*p* < 0.001) in all cases.

**Figure 7:**
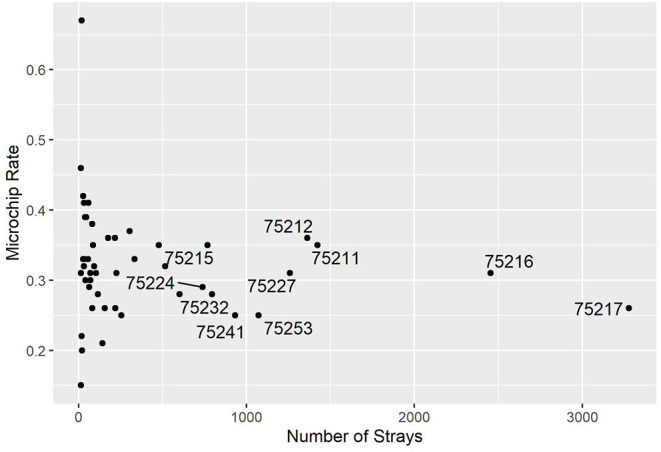
Microchip rate vs. number of strays by ZIP code. Highest-intake areas are labeled.

The distribution of microchip presence across town was also examined. For each ZIP code, the “microchip rate” was defined as the fraction of all stray intakes that were found with a microchip. Across the entire city, 30% of strays were found with microchips. The highest-intake ZIP code, 75217, was on the lower end of the microchip rate compared with other areas across Dallas, as shown in Figure 7. Since it is the largest intake ZIP code by a margin (alongside 75216, which was close to the average rate), it could be a good target to focus programs to promote microchip use. Other large ZIP codes are labeled.

## Discussion

While the results and figures are specific to DAS, they demonstrated the sort of insights that could arise from the tool. Starting with the big picture, the RTO rate was relatively high across the high-intake ZIP codes. From several conversations I had with other shelters and industry professionals, this is an anomaly. In another shelter that tried the tool, for example, there was a pronounced negative correlation between strays and RTO rate. The few large intake ZIP codes also accounted for a substantial amount of the shelter's overall intake, which suggested that even though the rates were at a strong starting point, these are the areas worth targeting for improving RTO rates even further. One area to investigate might be 75241, which had a lower overall and field-only RTO rate relative to other ZIP code with a similar stray intake size.

The most striking finding was that across Dallas, and particularly in the southern, high-intake ZIP codes, dogs rarely went far from home. Of all strays, 70% were found up to 1 mile away from home, and 42% were found within a block's range. The shelter expected something along these lines, but to see how close to home most dogs go, and have the data to back it up, was helpful. Plotting the median and 90th quantiles of distances also showed that the typical distances are similar across the city, but when it came to outliers, dogs found in northern ZIP codes tended to have gone farther from home—but usually still within the same ZIP code. This also aligned with the higher density of houses in southern neighborhoods. Since a successful RTO in the field saves a variety of resources that are given to every dog that gets brought into the shelter, these findings motivated investing in different programs that attempt to achieve RTOs within the neighborhood range. As a basic step, the shelter encourages community members, local lost and found groups, and field officers to further look for lost dogs within the neighborhood—perhaps an obvious suggestion, but the shelter now had data to effectively advocate for it. In addition, the shelter uses NextDoor, an information-exchange platform within a ZIP code, for posting lost pets within the area in which they were found. Another potential step is to equip field officers with posters to be hung around the block in which an animal is found.

The microchip enquiry raised another set of interesting findings. First, the results were in line with previous studies of RTO rates among microchipped and non-microchipped stray dogs, while also verifying the difference remains across physical characteristics, such as health condition. Although factors other than presence of a microchip could have contributed to these differences (for example, microchipping could be considered an indicator of responsible pet ownership), these results highlight the importance of microchips in reuniting owners and pets in Dallas and motivated more microchip-related programs. This was an encouraging result since there could be multiple challenges even if a microchip is present, including the chip not being registered or showing incorrect information. Further research and data collection are needed to characterize the reasons for RTO failure in cases of microchip presence. Finding that 75217, the highest-intake ZIP code, has among the lowest microchip rates helps to focus the efforts of microchip programs. One example that is being introduced is equipping field officers with microchips so that dogs found without one and are RTOed can undergo the process. The shelter is also looking into ways to reduce their microchip procedure fees, to lower the financial burden involved, and to encourage more owners who arrive at the shelter to use them.

The LOS results allow quantifying the time differences gained by RTOs compared with other outcome types. Of non-Field RTOs, 91% were reclaimed within the 5-day hold period, and 99% were reclaimed within 5 days after that period. Conversely, while 50% of dogs who ended up transferred or adopted stayed in the shelter an extra 1 or 2 days, respectively, 23 and 24% of dogs have stayed at least a week past the stray hold, and 10% stayed over 16 days in both non-RTO groups.

Moreover, plotting the results by Owner ZIP code shows that these rates are largely similar across the city—in other words, people who live across town take the same time to get their pets back from the shelter, which is good news. Had it not been the case, this sort of figure could motivate looking into ways to make the collection process easier for people who live farther from the shelter. Seeing that 91% of owners complete an RTO by 5 days affirms the shelter's choice to reduce the hold time to 5 days. For shelters with differing LOS averages by ZIP code, a potential experiment for improving RTO rates would be to vary these hold times and examine its effect on RTO rates in that area.

This analysis also has several limitations. First, it is just a starting point for spatially driven research to guide resource allocation. Using ZIP codes poses difficulties in focusing down on a specific area. Using higher-resolution data, such as Census tracts, would also enable integrating this with socioeconomic data, and one such direction is described in the next section. Another key layer of information that was not present here is the method of RTO and the RTO efforts attempted—was an RTO achieved due to a microchip, license record identification, or a Facebook group? Which attempts to identify an owner were made for successful and unsuccessful RTOs? Collecting this data, even for a short time, and integrating it with the existing analysis presented above would provide some further ideas for improving RTO rates. Relatedly, because there were no available data on microchip registration or correctness of detail, only a “microchip” vs. “no microchip” comparison could be made, rather than a more nuanced comparison, such as “microchip with correct data,” “microchip with incorrect data,” and “no microchip,” which would provide further insight into how RTO rates vary based on the microchip's data integrity. In addition, several statistical methods could be used to perform more nuanced analyses into some of the aspects of this study, such as LOS comparisons. Finally, as mentioned above, DAS's data were remarkably rich and complete, which enabled all sections of this analysis, but this may not be present for all shelters. Yet, while exact intake and outcome addresses may be harder to maintain, and microchip status is not always collected, all other types of data used are basic, which would allow many shelters to enjoy most of this tool. Hopefully, this paper also highlights the benefits of solid data integrity and encourages shelters to improve their data collection practice.

## Future Research

As more shelters have been interacting with the tool, new suggestions for visualizations and perspectives were added to the drawing table. As of writing this paper, a new page focused on euthanasia cases, broken down by intake ZIP codes, age groups, and intake conditions, was already added. Another set of improvements might come from a more convenient way of examining the differences in the findings above between different years, a first step of which was a time series that breaks down monthly intake and outcome patterns.

Another central inclusion involves demographic data. The live version of the tool includes Census data directly, such that shelter-level metrics can be assessed alongside human demographic data, such as median household income and percentage of foreign-born. For this purpose, all intake and outcome addresses were mapped onto Census tracts, and all other metrics were shifted from visualizing data by ZIP code to Census tracts, so that both demographic and shelter data are along the same spatial units. One implication of this transition for DAS was in designing their communications in a campaign launched in March 2021 to improve RTO rates. The stray and RTO metrics were used to choose focus areas as before, with Census level allowing a finer resolution than ZIP codes, and data about foreign languages spoken per Census tract guided the development of pamphlets and posters. The next step in this direction would be incorporating spatial data such as locations of pet food and medicine that would help illuminate some of the intake trends (for example, whether these indicators correlate with under-nourished intakes). The tool is planned to allow users to switch between ZIP codes and Census tracts to allow the benefits of both hierarchies.

The iterative development process of the tool has reaffirmed the notion that sparked it—shelters have shared interests. The tool currently spans across multiple aspects of a shelter's data—an overview of intake and RTO rates across town, the distances traveled by strays, the LOS for different outcome types, microchip trends and effects, and trends in euthanasia cases. Surely, not all shelters will find everything insightful. However, any new suggestion or feedback could be the beginning of an exciting change for another shelter—the scatter plot showing RTO rate vs. stray intake and the microchip inquiry are examples of development in response to suggestions or requests made by other shelters.

On the procedural level, the data still require preprocessing before being uploaded into the tool, for the reasons explained earlier: standardizing field values, calculating the distance traveled, fixing errors, and tuning the legend manuals. This might be an issue in attempting to scale the tool into many more shelters, but the processing time can currently take about one workday, so on the short-term, it is not prohibitive. In a later version, the tool could have a native way to upload a raw CSV file that would allow shelters to initiate preprocessing and get access to the tool within a day or two after the data are ready and loaded by the author. Expanding the computational infrastructure to support more shelters and automate some of the process is also possible, only subject to shelter interest and available resources; currently, using the tool is free of charge, and the author funds the hosting costs. After the data are cleaned and loaded, using the tool is intuitive *via* a web browser. The current version also includes a demo environment that any user can interact with featuring the DAS data.^1^

To conclude, I invite shelter directors and staff interested in further exploring their data, both those who found the above analysis compelling and others who wished to see something different—please reach out and join the process. Ultimately, I hope that the Databoard can continue to grow into a meaningful tool that could guide shelters' resource allocation, decision-making, and program planning and support their missions to improve the well-being of the animals and humans of their communities.^1^

## Data Availability Statement

The data analyzed in this study is subject to the following licenses/restrictions: The dataset is owned by Dallas Animal Services. Requests to access these datasets should be directed to tom.kremer@minerva.kgi.edu.

## Author Contributions

The author confirms being the sole contributor of this work and has approved it for publication.

## Conflict of Interest

The author declares that the research was conducted in the absence of any commercial or financial relationships that could be construed as a potential conflict of interest.

## Footnotes

^1^The current version of the tool with DAS's data can be found in the following URL: https://tomkremer.shinyapps.io/databoard/. Since this is a prototype, mind me for potential glitches.
